# Supplementary material for: The cost of attributing moral blame: Defensiveness and resistance to change when raising awareness to animal suffering in factory farming
Source: PLoS One. 2021 Aug 26;16(8):e0254375. doi: 10.1371/journal.pone.0254375 (PMC8389417; doi:10.1371/journal.pone.0254375)
Supplement: S1 File — (DOCX) [file pone.0254375.s001.docx]

**S1 File.**

Table of Contents

Manipulation material………………………………………………………………….2

Measures ………………………………………….......…………………………..…...5

Analyses with the preregistered attitudes measures…………………………………....7

Descriptive statistics for Study 2…….....…….......…………………………..…..…. .12

**Manipulation Materials**

English translation of the text, which differed between conditions.

Blame: We are all exposed to the lives of animals destined for food consumption - the way in which they are raised and transported for slaughter is done is done with complete disregard for the animal’s wellbeing. Today, most dairy and poultry farms are open to the public and are near residential areas. Who has not witnessed the trucks transporting chickens to the slaughter? It is evident to all that when you consume meat you are support the industry and its continuation. Even many rabbis now rule that the meat we consume is not kosher at all because it violates the prohibition of “tza’ar ba’aley chaim” [religious prohibition of cruelty to anima])." In a world where meat substitutes are healthy and plentiful, meat consumption serves self-indulgence only, and is no longer an existential need.

Absolve: Few of us are exposed to the lives of animals destined for food consumption, therefore most of us do not know that the way they are raised and transported is done with complete disregard for the animal’s wellbeing. Today, most dairy and poultry farms are hidden from public view, for fear of harming the industry’s profits. We tend to think that Kosher slaughter is moral and causes minimal suffering to the animal, however many rabbis now rule that the meat we consume is not kosher at all because it violates the prohibition of “tza’ar Ba’aley Chaim” [religious prohibition of cruelty to anima]). In addition, many countries (including Poland and Denmark) ban kosher slaughter for the suffering it causes.

Control: Animals destined for food consumption live in designated facilities throughout the country and are transported by various means to slaughter facilities. Today, dairy and poultry farms are located throughout the country, with the transportation of animals from the facilities to the slaughter being done mostly by trucks. Another source of meat that is sold in Israel is imported meat. About 55 tons, over half of the beef consumed in Israel, is imported from slaughterhouses in South America. There, kosher slaughter is carried out by rabbis authorized to do so. The Chief Rabbinate of Israel oversees the slaughter and kosher certification of meat imported into Israel in accordance with the Meat Law, which prohibits the import of non kosher meat to Israel.

Original material:

Blame:

**
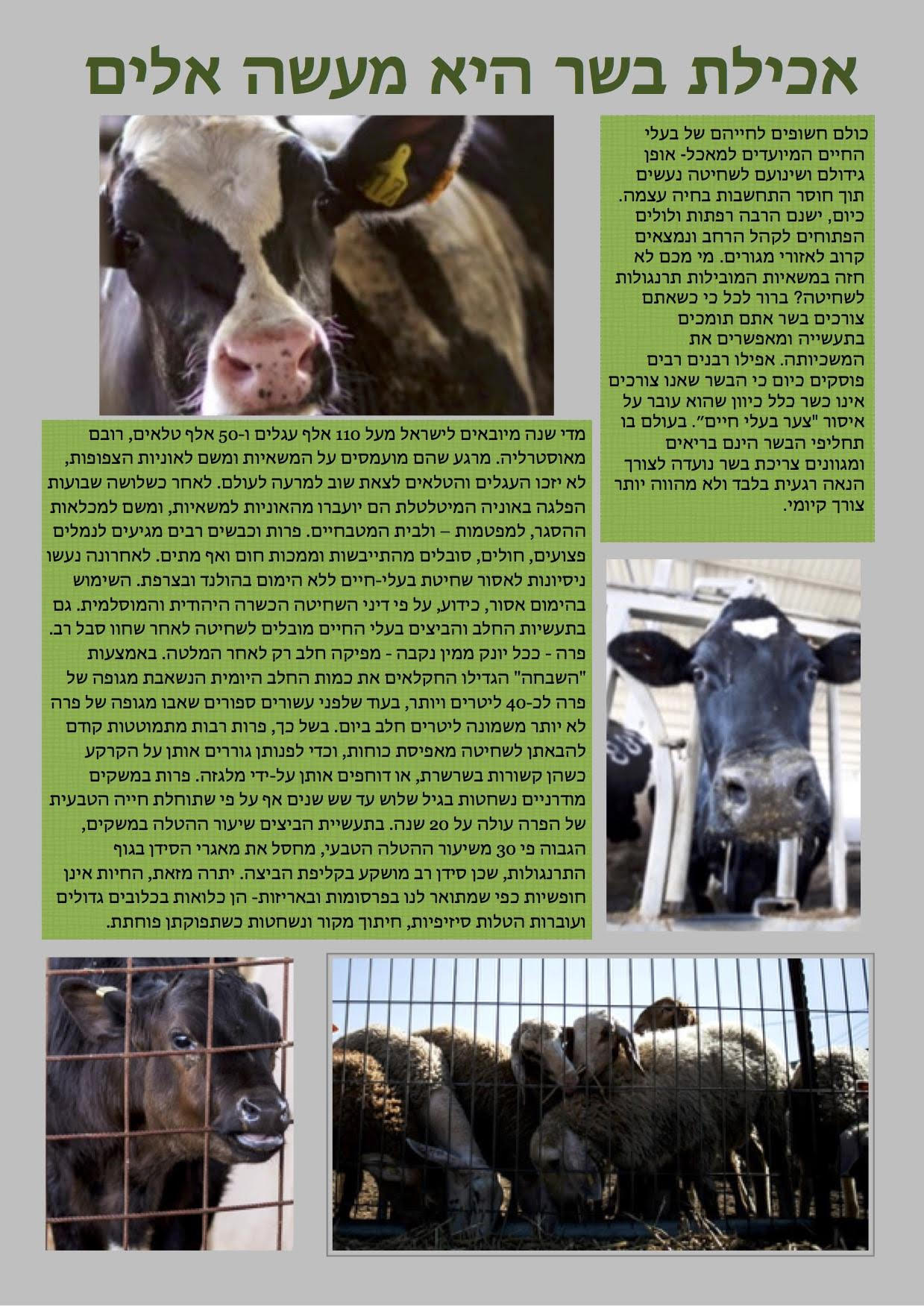
**

Absolve:


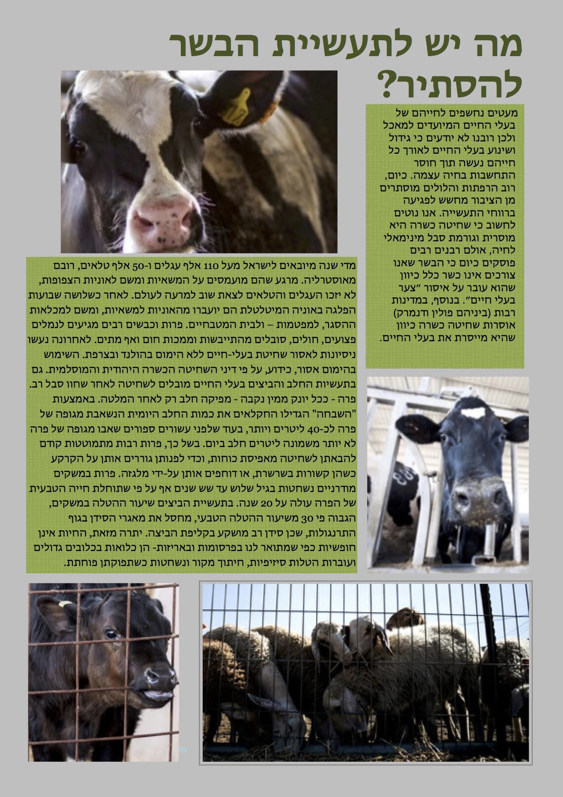


Control:

**
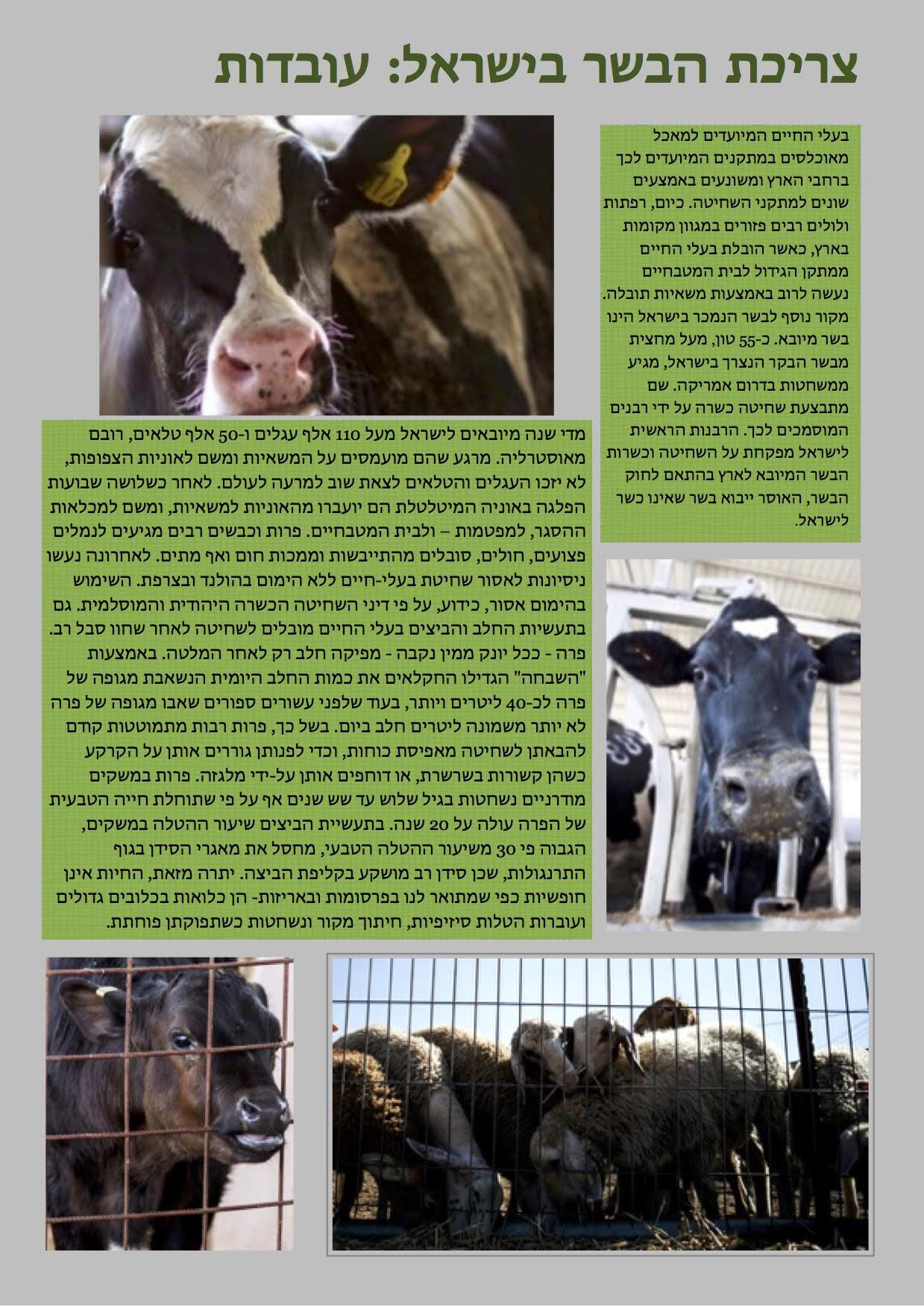
**

In addition to testing the effect of blaming (vs. absolving) messages, in Study 1, we had an exploratory goal of testing the effect of image type of attitudes and behavioral intentions towards veg*nism. Participants were presented with either a blaming or absolving text crossed with three sets of images capturing three degrees of animal suffering: animal suffering, neutral images, or happy animals. No differences were found based on image type. Data were, therefore, collapsed across image type and only differences between blame and absolve conditions were evaluated. In further studies, we used only th
kcnowlegments: taking part.andan that Michal Reifen Tagartion
his emotion...umaniraitan s and discussion lysis in Study 6.he neutral images.

**Measures**

**Positive attitudes towards veganism**

1. To what extent do you support veganism?
2. How important is veganism, in your opinion?
3. In your opinion, to what extent is it a positive thing for people to reduce their meat consumption? (item added to Study 3)

**Behavioral intentions towards veganism**

1. How much would you like to become vegan?
2. Would you like to receive an additional information about veganism?
3. Would you like to share information about veganism with your friends?
4. Are you willing to try meatless Monday?
5. To what extend would you be willing to reduce your meat consumption during the next month?

**Human superiority beliefs**

1. The life of an animal is just not of equal value as the life of a human being.
2. Animals are inferior to humans.
3. There is nothing unusual at all in the fact that humans dominate other animal species.
4. It is important that we treat other animal species more equally. (Recoded item)
5. In an ideal world, humans and animals would be treated on an equal basis” (reversed, item added to Study 3)
6. It is important that we treat other animal species more equally” (reversed, item added to Study 3)

**Efficacy beliefs**

1. If I wanted, I could become vegetarian
2. I am able to change my eating habits
3. If I decided to become vegan, I am sure I would succeed
4. Even if I wanted to, I could never give up meat

**Reactance**

1. I feel irritated that this message tried to make a decision for me
2. I feel angry that this message tried to pressure me
3. I feel annoyed that this message challenged my freedom to choose
4. I resent that this message tried to manipulate me

**Demoralization**

1. To what extent is veganism a moral question, in your opinion (reversed)

**4Ns**

1. It is only natural to eat meat
2. Our human ancestors ate meat all the time
3. It is unnatural to eat an all plant-based diet
4. Human beings are natural meat-eaters – we naturally crave meat
5. It is necessary to eat meat in order to be healthy
6. A healthy diet requires at least some meat
7. You cannot get all the protein, vitamins and minerals you need on an all plant- based diet
8. Human beings need to eat meat
9. It is normal to eat meat
10. It is abnormal for humans not to eat meat
11. Most people eat meat, and most people can’t be wrong
12. It is common for people to eat meat in our society, so not eating meat is socially offensive
13. Meat is delicious
14. Meat adds so much flavor to a meal it does not make sense to leave it out
15. The best tasting food is normally a meat-based dish (e.g., steak, chicken breast, grilled fish)
16. Meals without meat would just be bland and boring

**Vegan derogation**

To what extent do you think vegans are…

1. kind–mean
2. stupid–intelligent
3. healthy–unhealthy
4. judgmental– nonjudgmental
5. dirty–clean
6. weak– strong
7. humble–conceited
8. moral–immoral

**Analyses with the Preregistered Attitudes Measures**

Below are additional analyses using the attitudes towards veg*nism measure as preregistered. This measure originally included a moralization item that we later analyzed separately as a mediator (demoralization). In study 1 and 2, the original attitudes measure included the following items, *“To what extent do you support veganism?*” and “*How important is veganism, in your opinion?*” and the moralization item “*How much is veganism a moral question in your opinion?*”. In study 3, we added another item to the measure, “*In your opinion, to what extent is it a positive thing for people to reduce their meat consumption?*”. With or without the moralization item included, we found the same patterns of results across all studies.

**Study 1**

**The effect of condition (blame vs. absolve) on positive attitudes towards veg*nism.** Participants in the blame condition had less positive attitudes towards veg*nism (*M* = 4.53, *SD* = 2.37), compared with those in the absolve condition (*M* = 5.41, *SD* = 2.31), *t*(299) = 3.21 *p* < .001, *d* = 0.38.

**Defensiveness as a mediator between condition and positive attitudes towards veg*nism.** To test whether human superiority beliefs mediated the relationship between condition and attitudes, we ran a mediation analysis using Hayes’ PROCESS bootstrapping command with 5,000 iterations (model 4). Results indicated that participants who were blamed (vs. absolved) when provided with information about harm had stronger human superiority beliefs (*β* = .36, *SE* = .16, *t* = 2.25, *p* < .03). Human superiority beliefs led to less positive attitudes towards veg*nism (β = -.53, *SE* = .09, *t* = 5.64, *p* < .001). The effect of condition on attitudes (effect = -.87, *SE* = .27, *t* = 3.20, *p* < .002) was reduced after the mediator was considered in the model (effect = -.68, *SE* = .26, *t* = 2.61, *p* = .01), and the indirect effect of condition via human superiority beliefs (effect = -.19, SE = .09, 95% confidence interval (CI): (-.02, -.38) was significant, thus establishing mediation.

**Study 2**

**The effect of condition (blame vs. absolve vs. control) on attitudes towards veg*nism.** There was no significant difference between the control condition and the blame condition or the absolve condition on attitudes towards veg*nism.

**Study 3**

**The effect of condition (blame vs. absolve vs. control) on attitudes towards veg*nism.** Those who were blamed had significantly less positive attitudes towards veg*nism (*M* = 4.68, *SD* = 2.38) compared with those were absolved (*M* = 5.20, *SD* = 2.45), *t*(313) = 2.020, *p*= .02, *d* = .29). There was also a trend showing that those in the blame condition had less positive attitudes than those in the neutral control condition (*M* = 5.19, *SD* = 2.46), *t*(313)=1.46, *p*= .07, *d =*.02. There was no difference between the absolve and control conditions.

**Defensiveness as a mediator between blame (vs. absolve) and attitudes.** To test whether defense mechanisms mediated the relationship between blaming (vs. absolving) and attitudes towards veg*nism, we conducted mediational analysis using Hayes’ PROCESS bootstrapping command with 5,000 iterations (model 4). We found that reactance and efficacy beliefs mediated the effect of blaming (vs. absolving) on positive attitudes towards veg*nism. As this study also had a control group, we used the multicategorical function on Hayes’ Process that automatically creates dummy variables D1 (absolving vs. blaming) and D2 (absolving vs. control). We focused on the relationship between absolving and blaming on attitudes via three different defense mechanisms, as this was the mediated relationship we hypothesized. The analysis showed that the addition of reactance and efficacy beliefs reduced the relative total effect of blaming (vs. absolving) on positive attitudes towards veg*nism (effect =-.71, *SE* = .35, *p* = .02, 90% CI (-1.30, -.13) to non-significance (effect = -.28, *SE* = .32, 90% CI (-1.30, -.13) and the relative indirect effect through reactance (effect = -.21, *SE* = .11, 90% CI (-.41, -.04) and efficacy (effect = -.21, *SE* = .10, 90% CI (-.39, -.07) was significant. The model (see fig. S1) suggests that blame led to increased reactance and reduced efficacy beliefs, which in turn led to less positive attitudes towards veg*nism.


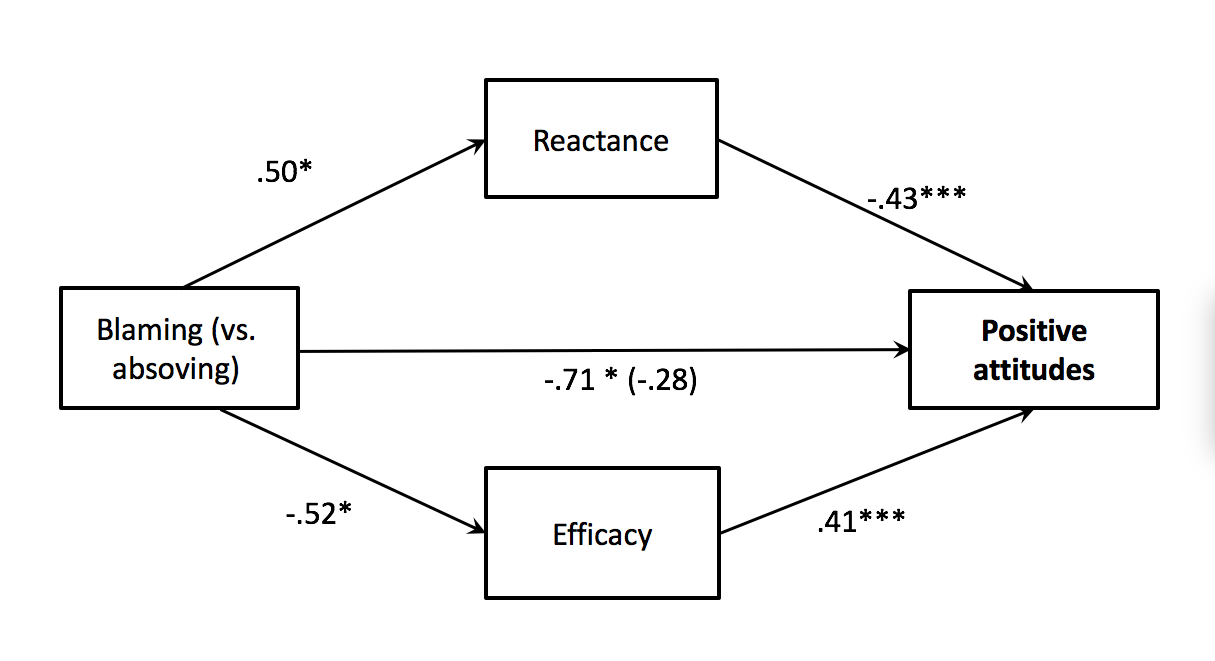


**Figure S1.**Models testing the effect of condition (blaming vs. absolving) on positive attitudes towards veg*nism through demoralization, reactance and efficacy beliefs for Study 3.

*Note*. Unstandardized coefficients displayed. **p*< .05. ***p*< .01. ****p*< .001, one tailed tests.

**Analysis showing Attitudes as a Mediator between Condition (Blame vs. Absolve) and Behavioral Intentions for Study 3**

For Study 3, we preregistered that positive attitudes towards veg*nism would mediate the relationship between condition (blame vs. absolve) and behavioural intentions in support of veg*nism, but as it is not central to our main research question, we report these analyses here and not in the main text. For this analysis, we used the original preregistered 4-item measure for attitudes towards veg*nism that included the moralization item.

To test whether attitudes mediated the relationship between blaming (vs. absolving) and behavior intentions in support of veg*nism we conducted mediational analysis using Hayes’ PROCESS bootstrapping command with 5,000 iterations (model 4). We used the original 4-item measure for attitudes that included the moralization item. We found that attitudes towards veg*nism mediated the effect of blaming (vs. absolving) on behavioral intentions towards veg*nism. As this study also had a control group, we used the multicategorical function on Hayes’ Process that automatically creates dummy variables D1 (absolving vs. blaming) and D2 (absolving vs. control). We focused on the relationship between absolving and blaming on behavioral intentions via attitudes, as this was the mediated relationship we hypothesized. The analysis showed that the addition of attitudes reduced the relative total effect of blaming (vs. absolving) on behavioral intentions (effect = -.80, *SE* = .33, *p* = .008, 90% CI (-1.34, -.26) to non-significance (effect = -.29, *SE* = .21, 90% CI (-.63, -.06) and the relative indirect effect through attitudes (effect = -.51, SE = .26, 90% CI (-.94, -.09) was significant. The model (see fig. S2) suggests that blame led to reduced positive attitudes towards veg*nism, which in turn led to less positive behavioral intentions towards veg*nism


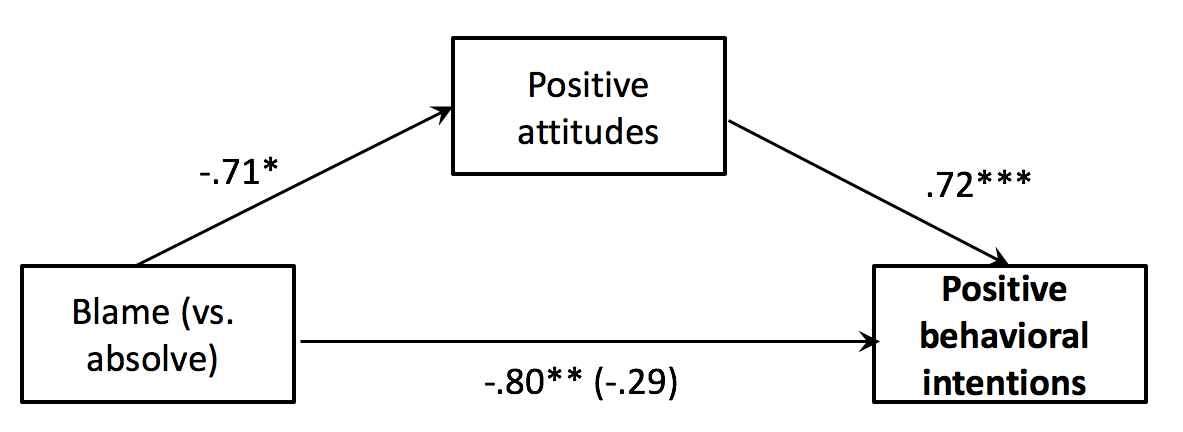


**Figure S2.**Models testing the effect of condition (blame vs. absolve) on positive behavioral intentions towards veg*nism through positive attitudes towards veg*nism for Study 3.

*Note*. Unstandardized coefficients displayed. **p*< .05. ***p*< .01. ****p*< .001, one tailed tests.

**Descriptive Statistics for Study 2**

Below, reported in table S1, are descriptive statistics for Study 2. These were not included in the main text, as ANOVAs were not significant.

|  |  | ***M(SD)*** | | | | |
| --- | --- | --- | --- | --- | --- | --- |
|  |  | **1-10 scale** | | | **1-7 scale** | |
|  | ***n*** | **Attitudes (2 items)** | **Behavioral intentions** | **De-**  **moralizaiton** | **Human superiority** | **Efficacy** |
| **Blame**  **Absolve**  **Control** | 213  245  231 | 3.67 (2.45)  3.78 (2.53)  4.00 (2.51) | 3.75 (2.33)  3.70 (2.49)  3.97 (2.52) | 6.15 (3.12)  6.57 (3.02)  6.05 (3.13) | 3.81 (1.37)  3.74 (1.46)  3.67 (1.44) | 4.26 (1.50)  4.37 (1.54)  4.35 (1.47) |

**Table S1.** Sample sizes, means, and standard deviations for Study 2 are reported per condition for outcome and mediator variables tested.
